# Oxidative Stress Biomarkers in the Relationship between Type 2 Diabetes and Air Pollution

**DOI:** 10.3390/antiox10081234

**Published:** 2021-07-30

**Authors:** Francesca Gorini, Laura Sabatino, Melania Gaggini, Kyriazoula Chatzianagnostou, Cristina Vassalle

**Affiliations:** 1 Institute of Clinical Physiology, Nazional Research Council, via Moruzzi 1, 56124 Pisa, Italy; laura.sabatino@ifc.cnr.it (L.S.); mgaggini@ifc.cnr.it (M.G.); 2 Fondazione CNR-Regione Toscana G. Monasterio, via Moruzzi 1, 54100 Pisa, Italy; zulachat@ftgm.it

**Keywords:** hyperglycemia, oxidative stress, pre-diabetes, type 2 diabetes, molecular mechanisms, air pollution, epigenetics, omics

## Abstract

The incidence and prevalence of type 2 diabetes have increased in the last decades and are expected to further grow in the coming years. Chronic hyperglycemia triggers free radical generation and causes increased oxidative stress, affecting a number of molecular mechanisms and cellular pathways, including the generation of advanced glycation end products, proinflammatory and procoagulant effects, induction of apoptosis, vascular smooth-muscle cell proliferation, endothelial and mitochondrial dysfunction, reduction of nitric oxide release, and activation of protein kinase C. Among type 2 diabetes determinants, many data have documented the adverse effects of environmental factors (e.g., air pollutants) through multiple exposure-induced mechanisms (e.g., systemic inflammation and oxidative stress, hypercoagulability, and endothelial and immune responses). Therefore, here we discuss the role of air pollution in oxidative stress-related damage to glycemic metabolism homeostasis, with a particular focus on its impact on health. In this context, the improvement of new advanced tools (e.g., omic techniques and the study of epigenetic changes) may provide a substantial contribution, helping in the evaluation of the individual in his biological totality, and offer a comprehensive assessment of the molecular, clinical, environmental, and epidemiological aspects.

## 1. Introduction

Data from the World Health Organization (WHO) evidenced that deaths from type 2 diabetes (T2D), which account for the majority of the total prevalence of diabetes, increased by 70% globally over the period of 2000–2019, especially in males (80% increase), more than doubled in the eastern Mediterranean [1], and this number is expected to increase by 25% in 2030 and 51% in 2045 [2]. However, a sticking point in this scenario is that one in two patients with diabetes is unaware that they are diabetic [2]. Another important aspect is that the risk of microvascular (e.g., retino-, nephro-, and neuropathy), and macrovascular (e.g., heart failure, peripheral arterial disease, and coronary heart disease) complications [3] is already significant long before the full-blown diagnosis of T2D, even in the presence of early and very mild dysglycemic alterations, in the so-called “prediabetes” phase.

In particular, this preT2D phase includes subjects with impaired fasting glucose and impaired glucose tolerance, according to the definitions most used for preT2D from the American Diabetes Association (ADA) and World Health Organization (WHO) (Table 1). Diagnostic tools for diagnosing preT2D or T2D include assessment of fasting glucose, two-hour glucose concentrations, and hemoglobin A1c [4].

PreT2D can subtly persist many years before the onset of overt T2D disease, resulting in the large epidemiological and clinical burden of this condition, which is estimated to affect 454 million people by 2030 and 548 million people by 2045 [4]. It is therefore crucial to identify subjects at risk of T2D as early as possible, and since chronic hyperglycemia is a strong stimulus for the production of free radicals and elevation of the oxidative stress state (e.g., increased generation of advanced glycation end products, mitochondrial dysfunction, proinflammatory and procoagulant effects, induction of apoptosis, vascular smooth-muscle cell proliferation, endothelial dysfunction and reduction of nitric oxide (NO) release, and activation of protein kinase C), the measurement of stress-related oxidative biomarkers could be useful [5].

Interestingly, many data have documented the adverse effects of environmental factors (e.g., air pollutants) on the onset and development of T2D through various exposure-induced mechanisms, including systemic inflammation and oxidative stress, hypercoagulability, endothelial and immune responses, and epigenetic modifications [6,7].

We are actually experiencing an era of development of new techniques, such as metabolomics and epigenetics, important for T2D pathobiology. Metabolomic techniques can reveal changes of metabolites and metabolic pathways before the onset of T2D and during the development of the disease, making it easier to understand the pathogenesis of T2D and improve its prediction, early diagnosis, and treatment. Likewise, epigenetic mechanisms appear to be involved in the modulation of gene expression, which influences the onset and progression of T2D and associated complications, an insight that helps to identify new biomarkers and drug targets in the future. However, studies evaluating omic (e.g., lipidomic or metabolomic) changes and epigenetic modifications exploring the relationships between air pollution and T2D are still scarce [8,9].

In this review, we discuss the role of the elusive relationship in the constant adjustment between pollution, oxidative stress, and T2D, highlighting the role of air pollution as a possible risk factor for glycemic abnormalities through the different cellular and molecular mechanisms that are linked to oxidative stress and inflammation, although thus far little considered in the clinical practice.

## 2. Diabetes and Air Pollution

Air pollution poses a considerable threat to health worldwide, being recognized as a leading contributing factor to the global disease burden [10,11]. According to WHO, in 2016, 91% of the world population lived in places where WHO air quality guidelines levels were not met, with low- and middle-income countries suffering the highest exposures [12]. Air pollution is a complex mixture of gases (e.g., nitrogen dioxide (NO_2_), carbon monoxide (CO), sulfur dioxide (SO_2_), and ozone (O_3_)) and particulate components (e.g., ≤10 μm in diameter (PM_10_); ≤2.5 μm (PM_2.5_)), whose sources and composition vary spatially and temporally [10,13].

It has been widely demonstrated that ambient air pollution, and especially PM, is associated with various chronic noncommunicable conditions such as cardiovascular disease (CVD) [14,15], neurological disorders [16], chronic obstructive pulmonary disease [17,18], and lung cancer [19,20]. More recently, growing evidence based on both epidemiological and experimental studies supports a potential role of air pollution in increasing the risk of insulin resistance (IR) and T2D [21,22].

So far, eight meta-analyses have analyzed the relationship of air pollution exposure with the occurrence, morbidity, and mortality of T2D (Table S1). Overall, a positive association risk has been documented for long-term exposure to air pollution and T2D, albeit a comparison of results sometimes remains difficult due to the difference in eligibility criteria for inclusion of the studies and methodology adopted [23]. Balti and co-authors (2014) included ten studies (five descriptive and five analytic) from developed countries (US, Canada, and Europe) to evaluate the effect estimates of exposure to nitrogen oxides, NO_2_, PM_2.5_ and PM_10_, and risk of T2D [24]. Diagnosis of T2D was based on a self-report or codes of the International Classification of Diseases, and most studies used single-pollutants models without considering the potential interaction between pollutants. The data revealed an increased occurrence of T2D of 13% and 11% for NO_2_ and PM_2.5_, respectively, and minimal heterogeneity was found in both analyses [24]. Consistently with these results, a meta-analysis of six cohort studies (three performed in the US and three in Europe) suggested an association between PM_2.5_ and incident T2D with an 11% increased risk [25]. In the meta-analysis by Wang et al. (2014), ten cohort studies performed in the US, Canada, and Europe were evaluated, investigating the associations of T2D risk with increments in PM_2.5_, PM_10_, or NO_2_ [26]. The incidence of T2D was significantly associated with long-term exposure to high levels of PM_2.5_, PM_10_ and NO_2_, with an increased risk by 28%, 15%, and 12%, respectively, while subgroup analyses by sex showed more pronounced effects in females, likely as a consequence of sex-related biological differences or gender-related behavioral or social differences (i.e., women spend most of their time at home, male participants tend to be more mobile) [25,26,27]. As evidenced in the meta-analysis by Balti et al. [24], there was a lack of data from developing or rapidly urbanizing countries, such as China, where the annual mean of PM_2.5_ is much higher than that measured in western countries [28] and the prevalence of T2D has been increasing sharply during the last three decades, with an overall prevalence of 10.9% in 2013 [29,30]. A subsequent meta-analysis comprising eight studies (five longitudinal, two cross-sectional, and one ecological, all conducted in western countries) reported an 11% and 8% increase in pooled risks for T2D per 10 μg/m^3^ increase of exposure to PM_2.5_ and NO_2_, respectively, confirming that females appear to be more susceptible than males [31]. In a meta-analysis of seven cohort studies, considering only long-term exposure to PM_2.5_, the risk of T2D increased by 25% with each 10 μg/m^3^ increase in PM_2.5_ [32]. Nonetheless, the major limitation of this study was estimation of the effect of a single pollutant without taking into account the combined impact of multiple exposures to air pollutants. More recently, Liu et al. (2019) conducted a meta-analysis of thirty studies (from North America, Europe, and other countries, most of them with cohort design) in order to estimate the pooled effect of air pollutant exposure on both the prevalence and incidence of T2D [33]. Regarding the prevalence of T2D, the meta-analysis showed a positive relationship with PM_2.5_, PM_10_, and NO_2_ (risk respectively of 9%, 12% and 5% per 10 μg/m^3^ of increase of each pollutant). Furthermore, no significant differences were detected in the subgroup analyses by country-level income and by sex for all three pollutants. On the other hand, the analysis of incidence revealed a significant association exclusively with long-term exposure to PM_2.5_, which resulted in a 10% risk of developing T2D. As stated by the authors, the observed discrepancy in the effects of PM_10_ and NO_2_ on T2D prevalence and incidence may be attributable to the presence of potential bias and confounding factors (e.g., exposure assessment, sample size, and environmental factors) in some studies [33]. A more comprehensive meta-analysis including 86 studies from 19 countries reported an increase in the prevalence and incidence of T2D following long-term exposure to PM_2.5_ and PM_10_, with comparable increases (range between 8 and 11%) [23]. As previously observed by Liu and co-workers (2019), NO_2_ exposure was significantly associated only with T2D prevalence (7% effect estimate for an increment of 10 μg/m^3^ in NO_2_) [33]. Long-term exposure to higher levels of PM_2.5_ and NO_2_, as assessed by four cohort studies, was also positively associated with higher T2D mortality. Although the small number of studies did not allow for a meta-analysis, cumulative evidence from case-crossover and time-series studies suggested a positive association of short-term exposure to air pollutants, in particular PM_2.5_, both with daily diabetes-caused mortality and all-cause mortality risk in diabetic subjects, who seemed more vulnerable to the adverse effects of air pollution than the nondiabetic population [23].

Two previous meta-analyses had assessed the relationship between major air pollutants and diabetes-associated mortality. Li et al. (2014) analyzed 12 studies (five time-series, five case-crossover, and two cohorts) from North America, Europe, and China showing a 12% increased risk in T2D-associated mortality per μg/m^3^ increment of PM_2.5_ and minor risks associated with high levels of PM_10_, NO_2_, and O_3_ [34]. In the other meta-analysis comprising 17 studies (with cohort, case-crossover, time-series, and case-control or cross-sectional design and most of them performed in North America and Europe), all the air pollutants examined (NO_2_, O_3_, PM_10_, SO_2_, and sulfate), with the exception of PM_2.5_, were positively, albeit weakly, associated with diabetes-related mortality or morbidity [35].

### 2.1. Mechanisms Underlying the Association between Diabetes and Air Pollution

Several potential biological mechanisms have been proposed to explain the link of T2D with air pollution-associated risk for CVD, including inflammation, endothelial dysfunction, and increased coagulability [7] (Table 2). Inflammation in response to PM exposure is a common mechanism, and experiments in animal models and in cell culture have reported increases in systemic and pulmonary levels of tumor necrosis factor alpha (TNFα), interleukin (IL)-6, and monocyte chemoattractant protein-1 (MCP-1) following PM exposure [36,37,38,39]. In humans, the results are more mixed, with some studies observing an increase in inflammatory markers in healthy subjects and in susceptible population groups exposed to fine and ultrafine particles [40,41,42,43], while other studies do not observe this [44,45,46]. In particular, molecular analysis revealed the activation of TLR4/NF-κB/COX-2, which led to the production of inflammatory cytokine in macrophages, following PM_2.5_ treatment [39].

Adipocytes are central in the control of energy balance and lipid homeostasis, and excess adipose tissue accumulation is a risk factor for both CVD and T2D [47]. While white adipose tissue (WAT) represents the primary site of energy storage with functions of triglyceride synthesis and glucose uptake, the physiological role of brown adipose tissue (BAT) is to metabolize fatty acids and generate heat [48]. Besides, adipose tissue plays an endocrine role by producing, in particular, the hormone leptin, which maintains energy homeostasis and adiposity, and adiponectin, whose reduced levels are implicated in the pathogenesis of IR in obesity and T2D [49]. Adipocytes secrete a number of inflammatory factors (e.g., TNFα, IL-1β, IL-6, and MCP-1) that directly affect insulin signaling [50]. In addition, increased levels of recruitment and/or activation of macrophages in visceral adipose tissue, which are a source of inflammatory factors, is a pathophysiologic hallmark of T2D in both humans and animal models [22,51]. Long-term exposure to PM_2.5_ has been shown to impair glucose tolerance and induce visceral inflammation/adiposity in a mouse model of diet-induced obesity [52]. Furthermore, PM_2.5_-exposed mice exhibited decreased glucose tolerance, systemic IR, IR in adipose tissue, liver, and skeletal muscle, increased F4/80+ macrophage recruitment/infiltration in the lung and WAT, and superoxide production and higher expression of 3-nitrotyrosine in BAT deposits, which is a marker of oxidative stress [53]. Reduced Akt phosphorylation in the liver, skeletal muscle, and WAT resulted in defective insulin signaling and consequent suppression of the insulin-stimulated glucose transporter translocation to the liver, which are fundamental events in the pathogenesis of IR/T2D [20,51]. Notably, in diet-induced obese rats (high-fat diet) but not in normal chow diet rats, exposure to PM_2.5_ increased IR, suggesting that obese subjects with IR may be a susceptible population to particulate air pollution [54].

A suggested biological pathway linking air pollution to T2D is endothelial dysfunction, which precedes IR resulting from reduced peripheral glucose uptake [55]. The vascular endothelium, a monolayer of cells lining the inner/luminal surface of blood vessels, represents a biologically active tissue regulating vascular tone and modulating vascular inflammation and injury mainly via the radical NO, thus endothelial dysfunction is associated with cardiovascular risk factors such as T2D, hypertension, hypercholesterolemia, and obesity [56,57]. Chronic exposure to PM_2.5_ has been shown to correlate with impaired endothelial function in both human [58,59,60,61] and experimental studies [62,63]. Specifically, PM_2.5_ can induce endothelial cytotoxicity by increasing endothelial cellular apoptosis via oxidative stress or autophagy, reducing endothelial cell migration and enhancing vascular endothelial permeability [57,62,64]. Also, PM_2.5_ exposure inhibits insulin-stimulated endothelial NO synthase (eNOS) phosphorylation, which leads to a decrease in eNOS activity and NO production, shifting the balance of vasomotor tone towards vasoconstriction and endothelial IR [64,65]. Indeed, altered endothelial function was more pronounced in subjects exposed to PM and exhibiting characteristics of an IR pattern (e.g., high body mass index, high glycosylated hemoglobin A1c, and low plasma adiponectin) [66].

Inflammation and endothelial dysfunction are also correlated with a hypercoagulability state. In particular, inflammation and the coagulation cascade are closely related, as acute inflammatory events activate the coagulation and fibrinolytic systems, while fibrin may directly influence inflammatory cell activities [67]. Furthermore, the proinflammatory cytokines TNFα, IL-1, and IL-6 can stimulate the release and expression of procoagulant molecules, such as von Willebrand factor antigen, tissue factor, and plasminogen activator inhibitor [68]. A recent meta-analysis evaluating the short- and long-term associations of PM with markers of inflammation and blood coagulation reported significant short-term associations of PM with TNFα and fibrinogen [69].

Another mechanism that links PM exposure with hepatic IR is the activation of endoplasmic reticulum (ER) stress, also called unfolded protein response (UPR), an intracellular signaling in response to the accumulation of unfolded or misfolded proteins [70]. As observed by Laing et al. (2010), ambient PM_2.5_ induced UPR signaling pathways in mice, relying on the production of reactive oxygen species, and triggering ER-stress induced apoptosis through the PERK-eIF2α-CHOP UPR branch in lung and liver tissues [64].

Mitochondrial capacity is considered a good indicator of insulin sensitivity, whereas the excessive glucose exposure or nutrient stress that occur in obesity and diabetes are associated with impaired glucose oxidation, reduced mitochondrial contents, and lowered rates of oxidative phosphorylation [71]. On the other hand, according to the new concept of “mitochondrial hormesis”, mitochondrial biogenesis (the generation of new mitochondria) and the activation of AMP-activated kinase (the main energy-sensing enzyme) enhance mitochondrial function and restore superoxide production, which in turn promote insulin secretion by pancreatic β cells, insulin sensitivity in skeletal muscle and liver, and improve endothelial function [72,73]. Additionally, long-term exposure to PM_2.5_ resulted in mitochondrial dysfunction, with a reduced mitochondrial number in WAT and mitochondrial size in BAT [53].

A number of different biomarkers related to oxidative stress and inflammation can be tested in the relationship between T2D and pollution. Some of them are, for example, C reactive protein, serum amyloid A, fibrinogen, cytokines (TNFα, interleukins), F2-isoprostanes, malondialdehyde, DNA breakdown products such as 8-hydroxy-2′-deoxyguanosine, advanced glycation end products, carbonylated proteins, and antioxidants or the total antioxidant status [74]. However, for most of these oxidative stress/inflammatory-related biomarkers and their methods of measurement, there are limitations that make routine use difficult, in particular the lack of analytical/clinical validation and technical challenges [75]. Many of these markers are also no-specific and, as such, linked to a number of pathophysiological conditions, and their significance can vary with the stage of the disease [75].

Moreover, there is no shared consensus on which biomarker/panel to use, due to the difficulties of choice related to the different types of redox reactions, tissue retention, and stage of cardiometabolic alterations [75]. In this setting, advances in high-throughput technologies (e.g., metabolomics) can help identify new critical biomarkers of risk and disease as well as potential therapeutic targets within the networks [76].

### 2.2. Omics and Epigenetics: A New Frontier in the Association between Diabetes and Air Pollution

**Figure 1:**
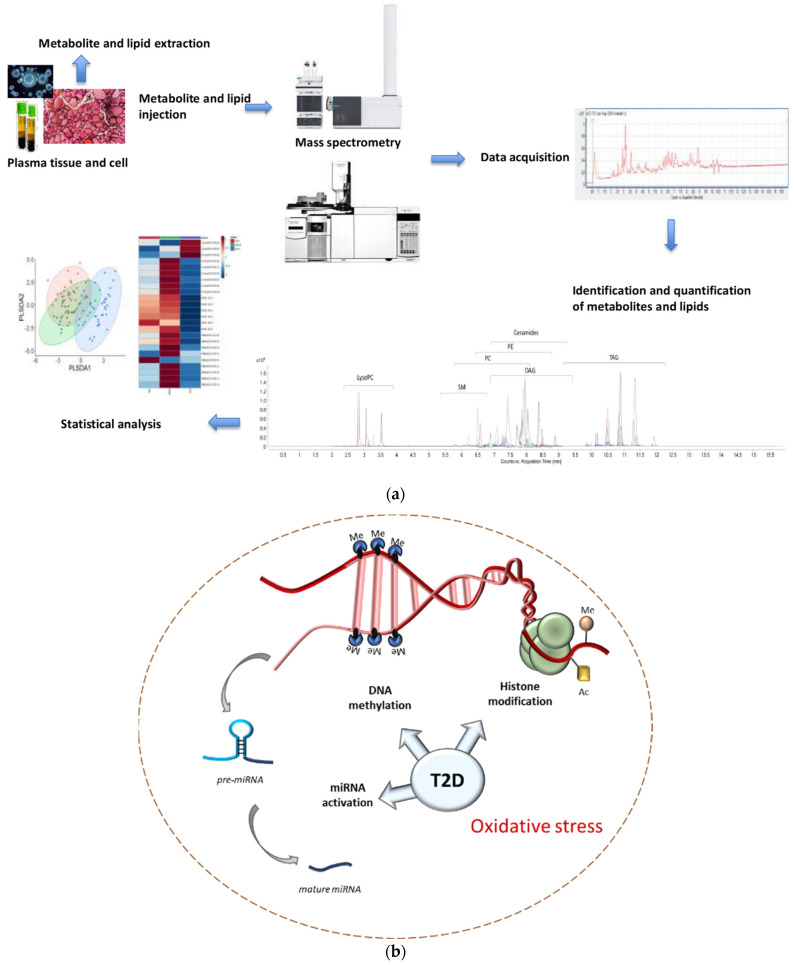
(**a**) Metabolites and lipids are extracted from tissues or biological fluids by several procedures (i.e., filch method, deproteinization) and injected into mass spectrometry instruments. Subsequently, the analysis of the chromatography acquired by the instruments leads to the identification and quantification of metabolites and lipids. Statistical analysis provides the significant changes regarding the detected metabolites/lipids. (**b**) Representation of the main epigenetic mechanisms in patients with type 2 diabetes: (1) Histone modification (in the example, acetylation and methylation), (2) DNA methylation, and (3) microRNA activation for post-transcriptional regulation.

The scientific community is currently experiencing an unprecedented era of emerging omic techniques and discoveries, which have allowed further insights into oxidative stress reactions, although they are always limited by the challenges related to data interpretation, the complexity of procedures, and the limited availability in routine laboratories. Previous studies have shown the crucial role of metabolomic approaches for the discovery of several metabolic biomarkers highly correlated with the onset and progression of T2D (Figure 1a) [77]. Among the metabolites, branched chain amino acids have been associated with the risk of metabolic disease, including T2D and nonalcoholic fatty liver disease [78,79]. Moreover, targeted metabolomic technologies have been used to analyze metabolites such as nitrotyrosine, associated with high levels of oxidative stress, in T2D subjects. In this context, Bala et al. recently demonstrated that four amino acids—cysteine, phenylalanine, tyrosine, and citrulline—correlated with the higher level of nitrotyrosine [80].

Accordingly, specific lipids could represent also reliable biomarkers of oxidative stress, IR, and hyperglycemia, being that T2D pathogenesis is characterized by an increase of tissue and plasma lipotoxicity [81,82]. In particular, through the plasma lipidomic profile, it is possible to evaluate lipidomic patterns associated with the risk of developing T2D [83,84,85].

New interesting insights are also emerging in the complex relationship between air pollution and T2D. In particular, a very recent study was conducted through a hepatic and plasma lipidomic analysis in mice exposed to ambient air pollution to evaluate lipid biomarkers related to PM_2.5_-mediated metabolic alterations such as IR, which were identified in molecules belonging to sphingomyelin-ceramide-glycosphingolipid pathways [8]. For instance, among glycerophospholipids, PI38:6, PI38:5, PI38:4, PI38:3, PI 40:5, and PI40:4 significantly increased in plasma, whereas lisocholine glycerophospholipid LPC18:3, LPC18:2, LPC18:0, LPC20:4, LPC22:6, and LPC22:5 significantly decreased. The plasma content of both total ceramides and sphingomyelins and specific classes of the two groups (as regards ceramides, 16:0, 18:0, 24:1, and 24:0 species; as regards sphingomyelins, 20:0, 21:1, 21:0, 22:1, 23:1, 23:0, 24:1, and 24:0 species) also increased in the plasma of mice exposed to PM_2.5_ [123]. In another experimental study, PM_2.5_ exposure was reported to affect plasma metabolome by inducing an increase in free fatty acid species and reducing phospholipid species, which in turn may contribute to vascular inflammation and loss of insulin sensitivity [9].

Another interesting development in this field is that relating to the study of epigenetic modifications. In fact, although most studies have focused on genes that affect cardiometabolic outcomes, nongenetic regulatory aspects are gaining growing attention in the last decade [86]. Epigenetic changes, which lead to alterations in gene expression through disturbances in the genome architecture that do not alter the DNA sequence, present themselves as distinctive features to a specific disease and offer novel opportunities for investigating the determining causes of the disease [87].

Epigenetic changes are generally classified into three main categories: DNA methylation, post-translational histone modifications, and noncoding RNA (ncRNA) [88] (Figure 1b).

In DNA methylation, a methyl group is covalently added to the carbon-5 position in the CpG dinucleotide sequence and represses gene activity by preventing the binding of transcription factors to gene promoters or allowing the recruitment of chromatin-modifying enzymes [89]. Unlike DNA methylation, the effects of histone modifications on gene expression can vary significantly, depending on the specific chemical modification [90]. Finally, ncRNA, including for example microRNAs (miRNAs) and long noncoding (lnc) RNAs, do not directly affect chromatin architecture but play an essential role in regulation of post-transcriptional gene expression [91].

A role of DNA methylation in oxidative stress leading to the onset or development of T2D was found for methylation variation at CpGs in the 3′-UTR of HIST1H4D and in the body of DVL1 [92]. Histone modification is also considered a key component of epigenetic modulation in T2D [93]. Finally, the discovery of ncRNAs has opened new insight into the role for these molecules as biomarkers of disease progression, and among these the miRNAs are ncRNAs of about 22 nucleotides, which regulate protein expression by binding mRNA and causing mRNA destabilization and/or translational repression [94]. Several studies identified different miRNAs implicated in pancreatic development, insulin secretion, and beta cell functional alteration [95]

Exposure to pollution may also induce epigenetic modifications, though most studies have examined endocrine disruptor chemicals (e.g., di-(2-ethylhexyl) phthalate, bisphenol A) rather than ultrafine particles and air gases, which are able to affect glucose metabolism and contribute to cardiometabolic disease [96]. Some data, however, support the association between PM exposure and epigenetic modifications. Long-term exposure to PM_2.5_ was associated with methylation changes in TNFα, enhancing its expression, and in the inflammatory TLR2 gene that could represent an epigenetic biomarker underlying the adverse health effects of air pollution [97]. Altered DNA methylation has been reported in oxidative and inflammatory genes such as those involved in “cytokine signaling” pathways, associated with both air pollution and the risk of CVD or obesity in the general population and obese subjects. Importantly, DNA changes in the methylation pattern of genes involved in oxidative stress, and inflammation may be detected several years before diagnosis, thus representing valuable preclinical biomarkers [98,99]. PM_2.5_ exposure has been also associated with modulation of the expression of different miRNAs (miR-21-5p, miR-187-3p, miR-146a-5p, miR-1-3p, and miR-199a-5p), ncRNAs (e.g., miR-3607-5p), circular RNA104250, and lncRNAuc001.dgp, which in turn may regulate cytokine expression, promoting an inflammatory response [100,101]. Furthermore, focusing on the relationship between air pollution and glucose abnormalities, short- and medium- term PM_2.5_ exposure resulted in higher fasting blood glucose in nondiabetic subjects, and this association was, at least in part, mediated by the decreased promoter methylation of the inflammatory gene ICAM-1 [102]. Interestingly, experimental results suggest that the chromatin remodeler SMARCA5 (SWI/SNF complex) was regulated in response to PM_2.5_ exposure, whereas the cessation of exposure was associated with a reversal of IR and restoration of chromatin accessibility and nucleosome positioning near transcription start sites, as well as a reversal of exposure-induced changes in the transcriptome, including SMARCA5 [103].

Therefore, although still at an early stage, these findings hold great promises for future studies, as they provide insights into further toxicological effects of PM_2.5_ that could explain the mechanisms triggering inflammation and subsequent oxidative stress in T2D and, in general, in cardiometabolic diseases.

## 3. Discussion

Although the topic needs further investigation and additional research is warranted to identify the link and mechanisms behind exposure to air pollution, the evidence is suggestive of an association between long-term exposure to higher air pollution levels, especially PM_2.5_ and NO_2_, and T2D development and mortality in adults [23,27]. As for the short-term effects of air pollution, if the overall results support a role for air pollutants in triggering early mortality in diabetic patients, these studies did not adjust the association for long-term air pollution exposure [23].

In the epidemiological studies, apart from a few common variables (e.g., age, sex, body mass index, smoking, and socioeconomic status) the confounding factors used in the adjusted risk estimates varied between the included studies, and along with differences in population demographics, this resulted in a high heterogeneity and, consequently, a reduced credibility of pooled evidence [23,24,27]. Likewise, different approaches to assessing exposure, i.e., land-use regression models to generate better exposure estimates or air monitors at fixed locations, instead of personal monitoring to collect information on all possible sources of air pollution, can provide a less accurate assessment of the risk of T2D [24,25,31]. Even a misclassification of outcome cannot be ruled out, since in some studies T2D cases resulted from self-reported diagnosis, in others they were based on hospital admission and diagnosis, and in still others diabetes status was measured directly from biomarkers, such as the oral glucose tolerance test, fasting plasma glucose concentration, insulin resistance, or medical records of antidiabetic medication use [23,33,104]. Furthermore, most of the findings came from high-income countries, whilst they were scarce from low- and middle-income countries, such as Pakistan and India [33]. Finally, based on the GRADE system for the scientific evaluation of quality studies, the assessment of the quality of epidemiological evidence derived from the published meta-analyses should be considered “poor” due to the observational nature of the studies included and the limitations described above [104].

Future studies should apply comparable models in assigning exposure to participants, in particular multipollutant models including lead, ozone, sulphate, polycyclic aromatic hydrocarbons, ultrafine particles, and possibly noise that was reported to correlate with traffic air pollution [31,105,106]. As has already been pointed out, a greater number of studies from developing countries are needed to provide a more accurate assessment of the influence of air pollution on T2D risk and the susceptibility of individuals with T2D to air pollution [34].

Among the numerous plausible mechanisms that have been hypothesized, the available data point to endothelial dysfunction, inflammation, and resulting oxidative stress as the major pathways involved in this relationship, albeit the exact mechanisms whereby exposure to air pollution is associated with an increased risk of diabetes remain to be elucidated [35,106].

Quantifying the role of air pollution in glycemic abnormalities and T2D is actually very complex for many reasons, and the assessment of exposure to air pollution is not included in the patient’s clinical evaluation, as it requires collection, analysis, and interpretation of data generally beyond the reach and competence of physicians. In this context, the contributions of metabolomics and lipidomics, as well as other new developments such as the study of epigenetic modifications, undoubtedly raise great expectations for further understanding pollution-induced alterations in T2D pathobiology and identifying novel key biomarkers, which can serve as targets for effective intervention strategies.

## 4. Conclusions

A huge body of evidence supports the potential role of long-term exposure to major air pollutants, primarily particulate matter, in the onset and development of T2D. Future prospective studies are warranted to better elucidate the effects of air pollution on T2D mortality. A comprehensive and comparable panel of biomarkers (including epigenetic changes, metabolites, and lipid markers) between studies can substantially help provide a more accurate assessment of this complex relationship and identify effective preventive strategies to reduce the burden of T2D and, in general, of cardiometabolic disease. However, this field is still in its infancy, and much more work is needed before these techniques can become effective clinical diagnostic tools. For these reasons, there are attempts to make these tools more useful in routine practice, such as proposing risk scores referring to the concentration of circulating ceramides for clinical application and cardiometabolic risk stratification in the clinical setting [107]. In any case, all these approaches together encourage a vision of personalized medicine focused on a specific care of the individual patient. Therefore, in the future, a multidisciplinary team with multiple skills will be essential to develop a personalized care strategy, responding to the peculiarities related to one’s own manifestation of risk or disease. In this scenario, the contribution of new advanced tools (e.g., *high-throughput* technologies) may be able to change the way we look at each patient, from the molecular to the clinical, environmental, and epidemiological levels, capturing the individual in his biological totality.

## Figures and Tables

**Table 1:** Pre-diabetes according to ADA and WHO criteria.

	ADA	WHO
IFG (Impaired fasting glucose)	5.6–7.0 mmol/L (100–125 mg/dL)	6.1–7.0 mmol/L (110–125 mg/dL)
IGT (Impaired glucose tolerance in the two-hour OGTT)	7.8–11.1 mmol/L (140–199 mg/dL)	7.8–11.1 mmol/L (140–199 mg/dL)
High risk HbA1c	39–46 mmol/mol (5.7–6.4%)	42–46 mmol/mol (6.0–6.4%)

ADA: American Diabetes Association; WHO: World Health Organization; HbA1c: Hemoglobin A1c; OGTT: Oral Glucose Tolerance Test.

**Table 2:** List of the molecules cited in the text as involved in the association between exposure to air pollutants and the risk of type 2 diabetes and their main biological actions.

Reference	Abbreviation	Molecule	Effects
[36,37,38,39]	TNFα	Tumor necrosis factor alpha	Promotion of acute inflammation, apoptosis
[36,37,38,39]	IL-6	Interleukin 6	Induction of the acute phase response, immune and hematopoietic activities
[68]	IL-1	Interleukin 1	Regulation of immune and inflammatory responses
[36,37,38,39]	MCP-1	Monocyte chemoattractant protein-1	Regulation of migration/infiltration of monocytes/macrophages
[39]	NF-κB	Nuclear factor kappa-light-chain-enhancer of activated B cells	Induction of the expression of various proinflammatory genes. Participation in inflammasome regulation. Regulation of the survival, activation and differentiation of innate immune cells and inflammatory T cells
[39]	COX-2	Cyclooxygenase 2	Production of the prostaglandins that contribute to pain, fever, and inflammation
[51]	Akt/PKB	Protein kinase N	Promotion of glucose metabolism, cell proliferation, transcription, migration, and apoptosis
[64,65]	NO	Nitric oxide	Control of vascular tone, dilation of blood vessels, reduction of blood pressure, inhibition of platelet aggregation (anti-thrombotic action)
[67]	-	Fibrin	Promotion of clot formation, fibrinolysis, cellular and matrix interactions, inflammation, and wound healing
[69]	-	Fibrinogen	During tissue and vascular injury, it is converted enzymatically by thrombin to fibrin
[68]	VWF	Von Willebrand factor	Promotion of platelet adhesion and, under high shear conditions, of platelet aggregation
[68]	TF	Tissue factor	Induction of blood coagulation
[68]	PAI-1	Plasminogen activator inhibitor 1	Inhibition of fibrinolysis

## Supplementary Materials

The following are available online at https://www.mdpi.com/article/10.3390/antiox10081234/s1, Table S1: Characteristics of included studies and main results reported in systematic reviews and meta-analyses evaluating the association between exposure to air pollutants and risk of T2D occurrence.

## Abbreviations

Ac	acetylation
BAT	brown adipose tissue
CVD	cardiovascular disease
IL	interleukin
IR	insulin resistance
MCP-1	monocyte chemoattractant protein-1
Me	methylation
miRNA	micro RNA
ncRNA	noncoding RNA
NO	nitric oxide
PM	particulate matter
T2D	type 2 diabetes
TNF-α	tumor necrosis factor alpha
UPR	unfolded protein response
WAT	white adipose tissue

## References

[1] WHO (World Health Organization) (2020). WHO Reveals Leading Causes of Death and Disability Worldwide: 2000–2019. https://www.who.int/news/item/09-12-2020-who-reveals-leading-causes-of-death-and-disability-worldwide-2000-2019.

[2] Saeedi P., Petersohn I., Salpea P., Malanda B., Karuranga S., Unwin N., Colagiuri S., Guariguata L., Motala A.A., Ogurtsova K. (2019). IDF Diabetes Atlas Committee. Global and regional diabetes prevalence estimates for 2019 and projections for 2030 and 2045: Results from the International Diabetes Federation Diabetes Atlas, 9th edition. Diabetes Res. Clin. Pract..

[3] Forouhi N.G., Wareham N.J. (2014). Epidemiology of diabetes. Medicine.

[4] Beulens J., Rutters F., Rydén L., Schnell O., Mellbin L., Hart H.E., Vos R.C. (2019). Risk and management of pre-diabetes. Eur. J. Prev. Cardiol..

[5] Papachristoforou E., Lambadiari V., Maratou E., Makrilakis K. (2020). Association of Glycemic Indices (Hyperglycemia, Glucose Variability, and Hypoglycemia) with Oxidative Stress and Diabetic Complications. J. Diabetes Res..

[6] Gangwar R.S., Bevan G.H., Palanivel R., Das L., Rajagopalan S. (2020). Oxidative stress pathways of air pollution mediated toxicity: Recent insights. Redox Biol..

[7] Brook R.D., Rajagopalan S., Pope C.A., Brook J.R., Bhatnagar A., Diez-Roux A.V., Holguin F., Hong Y., Luepker R.V., Mittleman M.A. (2010). American Heart Association Council on Epidemiology and Prevention; Council on the Kidney in Cardiovascular Disease, and Council on Nutrition, Physical Activity and Metabolism. Particulate matter air pollution and cardiovascular disease: An update to the scientific statement from the American Heart Association. Circulation.

[8] Li R., Wang Y., Hou B., Lam S.M., Zhang W., Chen R., Shui G., Sun Q., Qiang G., Liu C. (2020). Lipidomics insight into chronic exposure to ambient air pollution in mice. Environ. Pollut..

[9] Hill B.G., Rood B., Ribble A., Haberzettl P. (2021). Fine particulate matter (PM2.5) inhalation-induced alterations in the plasma lipidome as promoters of vascular inflammation and insulin resistance. Am. J. Physiol. Heart Circ. Physiol..

[10] Cohen A.J., Brauer M., Burnett R., Anderson H.R., Frostad J., Estep K., Balakrishnan K., Brunekreef B., Dandona L., Dandona R. (2017). Estimates and 25-year trends of the global burden of disease attributable to ambient air pollution: An analysis of data from the Global Burden of Diseases Study 2015. Lancet.

[11] Burnett R., Chen H., Szyszkowicz M., Fann N., Hubbell B., Pope C.A., Apte J.S., Brauer M., Cohen A., Weichenthal S. (2018). Global estimates of mortality associated with long-term exposure to outdoor fine particulate matter. Proc. Natl. Acad. Sci. USA.

[12] WHO (World Health Organization) (2018). Ambient (Outdoor) Air Pollution. Key Facts. https://www.who.int/news-room/fact-sheets/detail/ambient-(outdoor)-air-quality-and-health.

[13] Hamanaka R.B., Mutlu G.M. (2018). Particulate Matter Air Pollution: Effects on the Cardiovascular System. Front. Endocrinol..

[14] Cesaroni G., Forastiere F., Stafoggia M., Andersen Z.J., Badaloni C., Beelen R., Caracciolo B., de Faire U., Erbel R., Eriksen K.T. (2014). Long term exposure to ambient air pollution and incidence of acute coronary events: Prospective cohort study and meta-analysis in 11 European cohorts from the ESCAPE Project. BMJ.

[15] Pope C.A., Turner M.C., Burnett R.T., Jerrett M., Gapstur S.M., Diver W.R., Krewski D., Brook R.D. (2015). Relationships between fine particulate air pollution, cardiometabolic disorders, and cardiovascular mortality. Circ. Res..

[16] Fu P., Guo X., Cheung F.M.H., Yung K.K.L. (2019). The association between PM2.5 exposure and neurological disorders: A systematic review and meta-analysis. Sci. Total Environ..

[17] Berend N. (2016). Contribution of air pollution to COPD and small airway dysfunction. Respirology.

[18] Bloemsma L.D., Hoek G., Smit L.A.M. (2016). Panel studies of air pollution in patients with COPD: Systematic review and meta-analysis. Environ. Res..

[19] Malhotra J., Malvezzi M., Negri E., La Vecchia C., Boffetta P. (2016). Risk factors for lung cancer worldwide. Eur. Respir. J..

[20] Turner M.C., Andersen Z.J., Baccarelli A., Diver W.R., Gapstur S.M., Pope C.A., Prada D., Samet J., Thurston G., Cohen A. (2020). Outdoor air pollution and cancer: An overview of the current evidence and public health recommendations. CA Cancer J. Clin..

[21] Rajagopalan S., Brook R.D. (2012). Air pollution and type 2 diabetes: Mechanistic insights. Diabetes.

[22] Liu C., Ying Z., Harkema J., Sun Q., Rajagopalan S. (2013). Epidemiological and experimental links between air pollution and type 2 diabetes. Toxicol. Pathol..

[23] Yang B.Y., Fan S., Thiering E., Seissler J., Nowak D., Dong G.H., Heinrich J. (2020). Ambient air pollution and diabetes: A systematic review and meta-analysis. Environ. Res..

[24] Balti E.V., Echouffo-Tcheugui J.B., Yako Y.Y., Kengne A.P. (2014). Air pollution and risk of type 2 diabetes mellitus: A systematic review and meta-analysis. Diabetes Res. Clin. Pract..

[25] Park S.K., Wang W. (2014). Ambient Air Pollution and Type 2 Diabetes: A Systematic Review of Epidemiologic Research. Curr. Environ. Health Rep..

[26] Wang B., Xu D., Jing Z., Liu D., Yan S., Wang Y. (2014). Effect of long-term exposure to air pollution on type 2 diabetes mellitus risk: A systemic review and meta-analysis of cohort studies. Eur. J. Endocrinol..

[27] Dendup T., Feng X., Clingan S., Astell-Burt T. (2018). Environmental Risk Factors for Developing Type 2 Diabetes Mellitus: A Systematic Review. Int. J. Environ. Res. Public Health.

[28] WHO (World Health Organization) (2020). The Global Health Observatory. https://www.who.int/data/gho/data/indicators/indicator-details/GHO/concentrations-of-fine-particulate-matter-(pm2-5).

[29] Zuo H., Shi Z., Hussain A. (2014). Prevalence, trends and risk factors for the diabetes epidemic in China: A systematic review and meta-analysis. Diabetes Res. Clin. Pract..

[30] Zhou T., Liu X., Liu Y., Li X. (2019). Meta-analytic evaluation for the spatio-temporal patterns of the associations between common risk factors and type 2 diabetes in mainland China. Medicine.

[31] Eze I.C., Hemkens L.G., Bucher H.C., Hoffmann B., Schindler C., Künzli N., Schikowski T., Probst-Hensch N.M. (2015). Association between ambient air pollution and diabetes mellitus in Europe and North America: Systematic review and meta-analysis. Environ. Health Perspect..

[32] He D., Wu S., Zhao H., Qiu H., Fu Y., Li X., He Y. (2017). Association between particulate matter 2.5 and diabetes mellitus: A meta-analysis of cohort studies. J. Diabetes Investig.

[33] Liu F., Chen G., Huo W., Wang C., Liu S., Li N., Mao S., Hou Y., Lu Y., Xiang H. (2019). Associations between long-term exposure to ambient air pollution and risk of type 2 diabetes mellitus: A systematic review and meta-analysis. Environ. Pollut..

[34] Li C., Fang D., Xu D., Wang B., Zhao S., Yan S., Wang Y. (2014). Main air pollutants and diabetes-associated mortality: A systematic review and meta-analysis. Eur. J. Endocrinol..

[35] Janghorbani M., Momeni F., Mansourian M. (2014). Systematic review and metaanalysis of air pollution exposure and risk of diabetes. Eur. J. Epidemiol..

[36] Tamagawa E., Bai N., Morimoto K., Gray C., Mui T., Yatera K., Zhang X., Xing L., Li Y., Laher I. (2008). Particulate matter exposure induces persistent lung inflammation and endothelial dysfunction. Am. J. Physiol. Lung Cell. Mol. Physiol..

[37] Riva D.R., Magalhães C.B., Lopes A.A., Lanças T., Mauad T., Malm O., Valença S.S., Saldiva P.H., Faffe D.S., Zin W.A. (2011). Low dose of fine particulate matter (PM2.5) can induce acute oxidative stress, inflammation and pulmonary impairment in healthy mice. Inhal. Toxicol..

[38] Roper C., Chubb L.G., Cambal L., Tunno B., Clougherty J.E., Fattman C., Mischler S.E. (2017). Association of IL-6 with PM2.5 Components: Importance of Characterizing Filter-Based PM2.5 Following Extraction. Water Air Soil Pollut.

[39] Fu H., Liu X., Li W., Zu Y., Zhou F., Shou Q., Ding Z. (2020). PM2.5 Exposure Induces Inflammatory Response in Macrophages via the TLR4/COX-2/NF-κB Pathway. Inflammation.

[40] Törnqvist H., Mills N.L., Gonzalez M., Miller M.R., Robinson S.D., Megson I.L., Macnee W., Donaldson K., Söderberg S., Newby D.E. (2007). Persistent endothelial dysfunction in humans after diesel exhaust inhalation. Am. J. Respir. Crit. Care Med..

[41] Thompson A.M., Zanobetti A., Silverman F., Schwartz J., Coull B., Urch B., Speck M., Brook J.R., Manno M., Gold D.R. (2010). Baseline repeated measures from controlled human exposure studies: Associations between ambient air pollution exposure and the systemic inflammatory biomarkers IL-6 and fibrinogen. Environ. Health Perspect..

[42] Ostro B., Malig B., Broadwin R., Basu R., Gold E.B., Bromberger J.T., Derby C., Feinstein S., Greendale G.A., Jackson E.A. (2014). Chronic PM2.5 exposure and inflammation: Determining sensitive subgroups in mid-life women. Environ. Res..

[43] Dabass A., Talbott E.O., Venkat A., Rager J., Marsh G.M., Sharma R.K., Holguin F. (2016). Association of exposure to particulate matter (PM2.5) air pollution and biomarkers of cardiovascular disease risk in adult NHANES participants (2001–2008). Int. J. Hyg. Environ. Health.

[44] Mills N.L., Törnqvist H., Gonzalez M.C., Vink E., Robinson S.D., Söderberg S., Boon N.A., Donaldson K., Sandström T., Blomberg A. (2007). Ischemic and thrombotic effects of dilute diesel-exhaust inhalation in men with coronary heart disease. N. Engl. J. Med..

[45] Sullivan J.H., Hubbard R., Liu S.L., Shepherd K., Trenga C.A., Koenig J.Q., Chandler W.L., Kaufman J.D. (2007). A community study of the effect of particulate matter on blood measures of inflammation and thrombosis in an elderly population. Environ. Health.

[46] Teichert T., Vossoughi M., Vierkötter A., Sugiri D., Schikowski T., Schulte T., Roden M., Luckhaus C., Herder C., Krämer U. (2013). Association between traffic-related air pollution, subclinical inflammation and impaired glucose metabolism: Results from the SALIA study. PLoS ONE.

[47] Park K.W., Halperin D.S., Tontonoz P. (2008). Before they were fat: Adipocyte progenitors. Cell Metab..

[48] Spiegelman B.M., Flier J.S. (2001). Obesity and the regulation of energy balance. Cell.

[49] Waki H., Tontonoz P. (2007). Endocrine functions of adipose tissue. Annu. Rev. Pathol..

[50] Wellen K.E., Hotamisligil G.S. (2005). Inflammation, stress, and diabetes. J. Clin. Investig..

[51] Xu H., Barnes G.T., Yang Q., Tan G., Yang D., Chou C.J., Sole J., Nichols A., Ross J.S., Tartaglia L.A. (2003). Chronic inflammation in fat plays a crucial role in the development of obesity-related insulin resistance. J. Clin. Investig..

[52] Sun Q., Yue P., Deiuliis J.A., Lumeng C.N., Kampfrath T., Mikolaj M.B., Cai Y., Ostrowski M.C., Lu B., Parthasarathy S. (2009). Ambient air pollution exaggerates adipose inflammation and insulin resistance in a mouse model of diet-induced obesity. Circulation.

[53] Xu X., Liu C., Xu Z., Tzan K., Zhong M., Wang A., Lippmann M., Chen L.C., Rajagopalan S., Sun Q. (2011). Long-term exposure to ambient fine particulate pollution induces insulin resistance and mitochondrial alteration in adipose tissue. Toxicol. Sci..

[54] Yan Y.H., Chou C.C., Lee C.T., Liu J.Y., Cheng T.J. (2011). Enhanced insulin resistance in diet-induced obese rats exposed to fine particles by instillation. Inhal. Toxicol..

[55] Esposito K., Petrizzo M., Maiorino M.I., Bellastella G., Giugliano D. (2016). Particulate matter pollutants and risk of type 2 diabetes: A time for concern?. Endocrine.

[56] Daiber A., Steven S., Weber A., Shuvaev V.V., Muzykantov V.R., Laher I., Li H., Lamas S., Münzel T. (2017). Targeting vascular (endothelial) dysfunction. Br. J. Pharmacol..

[57] Münzel T., Gori T., Al-Kindi S., Deanfield J., Lelieveld J., Daiber A., Rajagopalan S. (2018). Effects of gaseous and solid constituents of air pollution on endothelial function. Eur. Heart J..

[58] Calderón-Garcidueñas L., Villarreal-Calderon R., Valencia-Salazar G., Henríquez-Roldán C., Gutiérrez-Castrellón P., Torres-Jardón R., Osnaya-Brizuela N., Romero L., Torres-Jardón R., Solt A. (2008). Systemic inflammation, endothelial dysfunction, and activation in clinically healthy children exposed to air pollutants. Inhal. Toxicol..

[59] Krishnan R.M., Adar S.D., Szpiro A.A., Jorgensen N.W., Van Hee V.C., Barr R.G., O’Neill M.S., Herrington D.M., Polak J.F., Kaufman J.D. (2012). Vascular responses to long- and short-term exposure to fine particulate matter: MESA Air (Multi-Ethnic Study of Atherosclerosis and Air Pollution). J. Am. Coll. Cardiol..

[60] Pope C.A., Bhatnagar A., McCracken J.P., Abplanalp W., Conklin D.J., O’Toole T. (2016). Exposure to Fine Particulate Air Pollution is Associated with Endothelial Injury and Systemic Inflammation. Circ. Res..

[61] Riggs D.W., Zafar N., Krishnasamy S., Yeager R., Rai S.N., Bhatnagar A., O’Toole T.E. (2020). Exposure to airborne fine particulate matter is associated with impaired endothelial function and biomarkers of oxidative stress and inflammation. Environ. Res..

[62] Hu H., Wu J., Li Q., Asweto C., Feng L., Yang X., Duan F., Duan J., Sun Z. (2016). Fine particulate matter induces vascular endothelial activation via IL-6 dependent JAK1/STAT3 signaling pathway. Toxicol. Res..

[63] Hou L., Zhang J., Zhang C., Xu Y., Zhu X., Yao C., Liu Y., Li T., Cao J. (2017). The injury of fine particulate matter from cooking oil fumes on umbilical cord blood vessels in vitro. Environ. Toxicol Pharmacol..

[64] Laing S., Wang G., Briazova T., Zhang C., Wang A., Zheng Z., Gow A., Chen A.F., Rajagopalan S., Chen L.C. (2010). Airborne particulate matter selectively activates endoplasmic reticulum stress response in the lung and liver tissues. Am. J. Physiol. Cell Physiol..

[65] Haberzettl P., O’Toole T.E., Bhatnagar A., Conklin D.J. (2016). Exposure to Fine Particulate Air Pollution Causes Vascular Insulin Resistance by Inducing Pulmonary Oxidative Stress. Environ. Health Perspect..

[66] Schneider A., Neas L., Herbst M.C., Case M., Williams R.W., Cascio W., Hinderliter A., Holguin F., Buse J.B., Dungan K. (2008). Endothelial dysfunction: Associations with exposure to ambient fine particles in diabetic individuals. Environ. Health Perspect..

[67] Luyendyk J.P., Schoenecker J.G., Flick M.J. (2019). The multifaceted role of fibrinogen in tissue injury and inflammation. Blood.

[68] Margetic S. (2012). Inflammation and haemostasis. Biochem. Med..

[69] Tang H., Cheng Z., Li N., Mao S., Ma R., He H., Niu Z., Chen X., Xiang H. (2020). The short- and long-term associations of particulate matter with inflammation and blood coagulation markers: A meta-analysis. Environ. Pollut..

[70] Zhang K., Kaufman R.J. (2008). From endoplasmic-reticulum stress to the inflammatory response. Nature.

[71] Wada J., Nakatsuka A. (2016). Mitochondrial Dynamics and Mitochondrial Dysfunction in Diabetes. Acta Med. Okayama.

[72] Dugan L.L., You Y.H., Ali S.S., Diamond-Stanic M., Miyamoto S., DeCleves A.E., Andreyev A., Quach T., Ly S., Shekhtman G. (2013). AMPK dysregulation promotes diabetes-related reduction of superoxide and mitochondrial function. J. Clin. Investig..

[73] Sharma K. (2015). Mitochondrial hormesis and diabetic complications. Diabetes.

[74] Menzel A., Samouda H., Dohet F., Loap S., Ellulu M.S., Bohn T. (2021). Common and Novel Markers for Measuring Inflammation and Oxidative Stress Ex Vivo in Research and Clinical Practice-Which to Use Regarding Disease Outcomes?. Antioxidants.

[75] Vassalle C. (2018). Oxidative stress and cardiovascular risk prediction: The long way towards a “radical” perspective. Int. J. Cardiol..

[76] Milburn M.V., Lawton K.A. (2013). Application of metabolomics to diagnosis of insulin resistance. Annu. Rev. Med..

[77] Yun J.H., Lee H.S., Yu H.Y., Kim Y.J., Jeon H.J., Oh T., Kim B.J., Choi H.J., Kim J.M. (2019). Metabolomics profiles associated with HbA1c levels in patients with type 2 diabetes. PLoS ONE.

[78] Sun Y., Gao H.Y., Fan Z.Y., He Y., Yan Y.X. (2020). Metabolomics Signatures in Type 2 Diabetes: A Systematic Review and Integrative Analysis. J. Clin. Endocrinol. Metab..

[79] Newgard C.B., An J., Bain J.R., Muehlbauer M.J., Stevens R.D., Lien L.F., Haqq A.M., Shah S.H., Arlotto M., Slentz C.A. (2009). A branched-chain amino acid-related metabolic signature that differentiates obese and lean humans and contributes to insulin resistance. Cell Metab..

[80] Bala C.G., Rusu A., Ciobanu D., Bucsa C., Roman G. (2021). Amino Acid Signature of Oxidative Stress in Patients with Type 2 Diabetes: Targeted Exploratory Metabolomic Research. Antioxidants.

[81] Zhu Y., Tsai M.Y., Sun Q., Hinkle S.N., Rawal S., Mendola P., Ferrara A., Albert P.S., Zhang C. (2018). A prospective and longitudinal study of plasma phospholipid saturated fatty acid profile in relation to cardiometabolic biomarkers and the risk of gestational diabetes. Am. J. Clin. Nutr..

[82] Ha C.Y., Kim J.Y., Paik J.K., Kim O.Y., Paik Y.H., Lee E.J., Lee J.H. (2012). The association of specific metabolites of lipid metabolism with markers of oxidative stress, inflammation and arterial stiffness in men with newly diagnosed type 2 diabetes. Clin. Endocrinol..

[83] Razquin C., Toledo E., Clish C.B., Ruiz-Canela M., Dennis C., Corella D., Papandreou C., Ros E., Estruch R., Guasch-Ferré M. (2018). Plasma Lipidomic Profiling and Risk of Type 2 Diabetes in the PREDIMED Trial. Diabetes Care.

[84] Yin X., Willinger C.M., Keefe J., Liu J., Fernández-Ortiz A., Ibáñez B., Peñalvo J., Adourian A., Chen G., Corella D. (2020). Lipidomic profiling identifies signatures of metabolic risk. EBioMedicine.

[85] Haus J.M., Kashyap S.R., Kasumov T., Zhang R., Kelly K.R., Defronzo R.A., Kirwan J.P. (2009). Plasma ceramides are elevated in obese subjects with type 2 diabetes and correlate with the severity of insulin resistance. Diabetes.

[86] Brunet A., Berger S.L. (2014). Epigenetics of aging and aging-related disease. J. Gerontol. A Biol. Sci. Med. Sci..

[87] Berger S.L., Kouzarides T., Shiekhattar R., Shilatifard A. (2009). An operational definition of epigenetics. Genes Dev..

[88] Handy D.E., Castro R., Loscalzo J. (2011). Epigenetic modifications: Basic mechanisms and role in cardiovascular disease. Circulation.

[89] Kohli R.M., Zhang Y. (2013). TET enzymes, TDG and the dynamics of DNA demethylation. Nature.

[90] Shahbazian M.D., Grunstein M. (2007). Functions of site-specific histone acetylation and deacetylation. Annu. Rev. Biochem..

[91] Gurha P., Marian A.J. (2013). Noncoding RNAs in cardiovascular biology and disease. Circ. Res..

[92] Hedman Å.K., Zilmer M., Sundström J., Lind L., Ingelsson E. (2016). DNA methylation patterns associated with oxidative stress in an ageing population. BMC Med. Genom..

[93] Sims R.J., Reinberg D. (2008). Is there a code embedded in proteins that is based on post-translational modifications?. Nat. Rev. Mol. Cell Biol..

[94] Krol J., Loedige I., Filipowicz W. (2010). The widespread regulation of microRNA biogenesis, function and decay. Nat. Rev. Genet..

[95] Tang X., Tang G., Ozcan S. (2008). Role of microRNAs in diabetes. Biochim. Biophys. Acta.

[96] Imam M.U., Ismail M. (2017). The Impact of Traditional Food and Lifestyle Behavior on Epigenetic Burden of Chronic Disease. Glob. Chall..

[97] Ramos-Lopez O., Milagro F.I., Riezu-Boj J.I., Martinez J.A. (2021). Epigenetic signatures underlying inflammation: An interplay of nutrition, physical activity, metabolic diseases, and environmental factors for personalized nutrition. Inflamm. Res..

[98] Fiorito G., Vlaanderen J., Polidoro S., Gulliver J., Galassi C., Ranzi A., Krogh V., Grioni S., Agnoli C., Sacerdote C. (2018). Oxidative stress and inflammation mediate the effect of air pollution on cardio- and cerebrovascular disease: A prospective study in nonsmokers. Environ. Mol. Mutagen..

[99] Cantone L., Iodice S., Tarantini L., Albetti B., Restelli I., Vigna L., Bonzini M., Pesatori A.C., Bollati V. (2017). Particulate matter exposure is associated with inflammatory gene methylation in obese subjects. Environ. Res..

[100] Chen R., Li H., Cai J., Wang C., Lin Z., Liu C., Niu Y., Zhao Z., Li W., Kan H. (2018). Fine Particulate Air Pollution and the Expression of microRNAs and Circulating Cytokines Relevant to Inflammation, Coagulation, and Vasoconstriction. Environ. Health Perspect..

[101] Li X., Jia Y., Nan A., Zhang N., Zhou H., Chen L., Pan X., Qiu M., Zhu J., Zhang H. (2020). CircRNA104250 and lncRNAuc001.dgp.1 promote the PM2.5-induced inflammatory response by co-targeting miR-3607-5p in BEAS-2B cells. Environ. Pollut..

[102] Peng C., Bind M.C., Colicino E., Kloog I., Byun H.M., Cantone L., Trevisi L., Zhong J., Brennan K., Dereix A.E. (2016). Particulate Air Pollution and Fasting Blood Glucose in Nondiabetic Individuals: Associations and Epigenetic Mediation in the Normative Aging Study, 2000–2011. Environ. Health Perspect..

[103] Rajagopalan S., Park B., Palanivel R., Vinayachandran V., Deiuliis J.A., Gangwar R.S., Das L., Yin J., Choi Y., Al-Kindi S. (2020). Metabolic effects of air pollution exposure and reversibility. J. Clin. Investig..

[104] Dimakakou E., Johnston H.J., Streftaris G., Cherrie J.W. (2018). Exposure to Environmental and Occupational Particulate Air Pollution as a Potential Contributor to Neurodegeneration and Diabetes: A Systematic Review of Epidemiological Research. Int. J. Environ. Res. Public Health.

[105] Khan J., Ketzel M., Kakosimos K., Sørensen M., Jensen S.S. (2018). Road traffic air and noise pollution exposure assessment—A review of tools and techniques. Sci. Total Environ..

[106] Lim C.C., Thurston G.D. (2019). Air Pollution, Oxidative Stress, and Diabetes: A Life Course Epidemiologic Perspective. Curr. Diab. Rep..

[107] Hilvo M., Vasile V.C., Donato L.J., Hurme R., Laaksonen R. (2020). Ceramides and Ceramide Scores: Clinical Applications for Cardiometabolic Risk Stratification. Front. Endocrinol. (Lausanne).

