# Supplementary material for: Oxidative Stress Biomarkers in the Relationship between Type 2 Diabetes and Air Pollution
Source: Antioxidants (Basel). 2021 Jul 30;10(8):1234. doi: 10.3390/antiox10081234 (PMC8388875; doi:10.3390/antiox10081234)
Supplement: Supplementary file 1 [file antioxidants-10-01234-s001.zip › antioxidants-1324394-supplementary.pdf]

Table S1. Characteristics of included studies and main results reported in systematic reviews and meta-analyses evaluating the association between exposure to air pollutants and T2D risk

Study design	Study period/ Countries	Sample size/ Age	Case number	Outcome assessment	Pooled risk estimates (95%CI)	Confounders	Reference
5 cohort,<br>4 cross-sectional,<br>1 ecological	1989-2010<br>Canada, US, Denmark, The Netherlands, Germany, Switzerland	506,157<br>≥21 years	34,152	Self-reported diabetes, doctor-diagnosed T2D, hospital discharge/admission for T2D, anti-diabetic medications, elevated FBG or RBG levels, elevated plasma glucose levels after OGTT, T2D symptoms	T2D incidence<br>*NO_2*<br>HR: 1.13 (1.01-1.22)<br>*$PM_{2.5}$*<br>HR: 1.11 (1.03-1.20)	Age, sex, race, BMI, waist-to-hip ratio, obesity, hypertension, hypercholesterolemia, previous myocardial infarction, high hsCRP, dyslipidemia, lifestyle characteristics, physical activity, fruit consumption, fat consumption, diet, smoking, alcohol consumption, marital status, educational level, health insurance, per capita income, number of people in the household, environmental tobacco smoke, noise, heating with fossil fuels, workplace exposure with dust/fumes, extreme temperatures, neighborhood socioeconomic index, population density, latitude, urban residency, state of residence, season, calendar year	24
6 prospective cohort	1989-2010<br>Canada, US, Germany, Denmark	209,057<br>21-69 years	14,029	National diabetes data/registers, self-reported physician diagnosis, elevated plasma glucose levels, T2D symptoms, hypoglycemic medications	T2D incidence<br>*$PM_{2.5}$*<br>HR: 1.11 (1.03-1.19)<br>for a 10 μg/m^3 increase	Age, sex, race, BMI, waist-to-hip ratio, hypertension, fruit, fat, diet, physical activity, education, smoking, alcohol, income, heating w/fossil fuels, workplace exposure w/dust fumes, extreme temperature, neighborhood socioeconomic index, residence state, urban residency, season, calendar year	25
10 prospective cohort	1989-2010<br>Canada, US, Germany, Denmark, Switzerland	2,371,907<br>≥21 years	21,095	Hospital admission, mortality from diabetes, intake of any anti-diabetic medication, reimbursement for chiropody due to diabetes, self-report of doctor diagnosed T2D, glucose blood test, elevated HbA1c	T2D incidence<br>*$PM_{2.5}$*<br>RR: 1.28 (1.06-1.55)<br>Males RR: 1.30 (0.95-1.77)<br>Females RR: 1.28 (1.10-1.50)<br>per 10 μg/m^3 increment<br>*PM_{10}*<br>RR: 1.15 (1.02-1.30)<br>Males RR: 1.03 (0.96-1.10)<br>Females RR: 1.09 (1.02-1.15)<br>per 10 μg/m^3 increment<br>*NO_2*<br>RR: 1.12 (1.02-1.23)<br>Males RR: 1.03 (0.96-1.10)<br>Females RR: 1.09 (1.02-1.15)	Age, sex, race/ethnicity, BMI, waist-to-hip ratio, hypertension, hsCRP, history of T2D, dyslipidemia, cholesterol, physical activity, fruit consumption, fat consumption, diet, smoking, alcohol consumption, educational level, occupational level, income, number of people in the household, environmental tobacco smoke, noise, annual mean concentration of $PM_{2.5}$, workplace exposure with dust/fumes, extreme temperatures, neighborhood socioeconomic index, city of residence, season, calendar year	26

Study design	Study period/ Countries	Sample size/ Age	Case number	Outcome assessment	Pooled risk estimates (95%CI)	Confounders	Reference
					per 10 μg/m³ increment		
5 longitudinal, 2 cross-sectional, 1 ecological	1989-2010 Canada, US, Denmark, The Netherlands, Germany	312,233 ≥21 years	20,372	Self-reported diabetes, physician diagnosed T2D, county level detection T2D health survey data, national diabetes data/registers, laboratory-based diagnosis for undetected cases	T2D incidence<br>*$PM_{2.5}$*<br>RR: 1.10 (1.02-1.18)<br>Males RR: 1.04 (0.93-1.17)<br>Females RR: 1.14 (1.03-1.26)<br>per 10 μg/m³ increment<br>*NO_2*<br>RR: 1.08 (1.00-1.18)<br>Males RR: 0.99 (0.93-1.07)<br>Females RR: 1.15 (1.05-1.27)<br>per 10 μg/m³ increment	Age, sex, race, BMI, waist-to-hip ratio, obesity, hypertension, hypercholesterolemia, previous myocardial infarction, lifestyle characteristics, physical activity, fruit consumption, fat consumption, diet, smoking, alcohol consumption, marital status, educational level, health insurance, per capita income, number of people in the household, environmental tobacco smoke, heating with fossil fuels, workplace exposure with dust/fumes, extreme temperatures, neighborhood socioeconomic index, population density, latitude, urban residency, state of residence, season, calendar year	31
7 prospective cohort	1989-2013 Canada, US, Germany	2,339,859 25-84 years	23,639	National diabetes data/registers, health administrative data, mortality from diabetes, hospital admission with a diagnosis of diabetes, self-report of doctor-diagnosed of T2D, elevated fasting glucose level, treatment with hypoglycemic medication, diabetes symptoms	T2D incidence<br>*$PM_{2.5}$*<br>RR: 1.25 (1.10-1.43)<br>per 10 μg/m³ increment	Age, sex, race/ethnicity, BMI, hypertension, history of T2D, lifestyle characteristics, physical activity, diet, smoking, alcohol consumption, annual mean concentration of $PM_{2.5}$, educational level, occupational level, state of residence, season, calendar year, number of people in the household	32
17 cohort, 12 cross-sectional, 1 ecological	1999-2014 Canada, US, Italy, France, Germany, Denmark, Switzerland, The Netherlands, China, Korea, Australia	32,455,963 ≥18 years	6,529,770	Health administrative data, self-report, screening, physician examination/diagnosed T2D, elevate fasting glucose or HbA1c, 2-h OGTT, questionnaires, antidiabetes medications, hospitalization records, health survey data, national diabetes data/registers	T2D prevalence<br>*$PM_{2.5}$*<br>OR: 1.09 (1.05-1.13)<br>Males OR: 1.10 (1.05-1.15)<br>Females OR: 1.14 (1.05-1.24)<br>*PM_{10}*<br>OR: 1.12 (1.06-1.19)<br>Males OR: 1.14 (1.07-1.21)<br>Females OR: 1.10 (0.97-1.25)<br>*NO_2*<br>OR: 1.05 (1.03-1.08)<br>Males OR: 1.05 (1.01-1.0)<br>Females OR: 1.03 (1.00-1.06)	Age, sex, place of birth, race, BMI, waist-to-hip ratio, obesity, hypertension, family, history of T2D, hypercholesterolemia, previous myocardial infarction, stroke, ischemic heart disease, congestive heart failure, chronic lung disease, asthma, COPD, cancer, estimated glomerular filtration rate, high hsCRP, dyslipidemia, medication taken, use of an angiotensin-converting enzyme inhibitor or angiotensin receptor blocker, number of admissions to hospital before beginning of follow-up, how many times serum creatinine was measured before	33

Study design	Study period/ Countries	Sample size/ Age	Case number	Outcome assessment	Pooled risk estimates (95%CI)	Confounders	Reference
					T2D incidence<br>*$PM_{2.5}$*<br>HR: 1.10 (1.04-1.16)<br>Males HR: 1.05 (1.01-1.08)<br>Females HR: 1.12 (1.03-1.21)<br>*PM_{10}*<br>HR: 1.05 (0.98-1.13)<br>Males HR: 1.08 (0.92-1.27)<br>Females HR: 1.07 (0.97-1.19)<br>*NO_2*<br>HR: 1.02 (0.99-1.05)<br>Males HR: 0.98 (0.96-1.00)<br>Females HR: 1.05 (0.99-1.11)	beginning of follow-up lifestyle characteristics, physical activity, fruit consumption, fat consumption, diet, smoking status and pack-years,, alcohol/sugar-sweetened soft drinking consumption, marital status, educational level, occupational status/unemployment rate, health insurance, per capita/household income, number of people in the household, environmental tobacco smoke, traffic noise annoyance ozone, heating with indoor combustion, workplace exposure with dust/fumes, type of heating energy, type of cooking energy, extreme temperatures, neighborhood socioeconomic index, census track data on median household income, percent of households living below the poverty line, neighborhood level recent immigrants, population density, prevalence of age≥65, latitude, urban residency, state of residence, region, urbanity, season, calendar year	
20 cohort,<br>21 cross-sectional,<br>2 ecological, 1 case-crossover,<br>1 time-series	1991-2015<br>Canada, US, Italy, France, Germany, Denmark, Switzerland, The Netherlands, United Kingdom, China, Korea, Taiwan, Australia, Saudi Arabia, Iran	32,798,442	6,518,915	Health administrative data, self-reported or registered, self-report of doctor-diagnosed diabetes, screening, physician examination/diagnosed T2D, elevate fasting glucose or HbA1c, 2-h OGTT, questionnaires, antidiabetes medications, hospitalization records, health survey data, national diabetes data/registers	T2D prevalence<br>*$PM_{2.5}$*<br>OR: 1.10 (1.04-1.17)<br>*PM_{10}*<br>OR: 1.11 (1.00-1.22)<br>*NO_2*<br>OR: 1.01 (0.99-1.02)<br>per 10 μg/m³ increment<br><br>T2D incidence<br>*$PM_{2.5}$*<br>HR: 1.08 (1.04-1.12)<br>*PM_{10}*<br>HR: 1.10 (1.03-1.17)<br>*NO_2*<br>HR: 1.07 (1.04-1.11)<br>per 10 μg/m³ increment	Age, sex, place of birth, race, BMI, waist-to-hip ratio, obesity, diastolic blood pressure, hypertension, family, history of T2D, hypercholesterolemia, previous myocardial infarction, stroke, ischemic heart disease, congestive heart failure, chronic lung disease, asthma, COPD, cancer, estimated glomerular filtration rate, high hsCRP, dyslipidemia, medication taken, use of an angiotensin-converting enzyme inhibitor or angiotensin receptor blocker, number of admissions to hospital before beginning of follow-up, how many times serum creatinine was measured before beginning of follow-up lifestyle characteristics, physical activity, fruit consumption, fat consumption, diet, vegetable/fruit diet pattern, meat/fried food diet pattern, smoking status and pack-years, alcohol/sugar-sweetened soft drinking consumption, marital status, educational level, occupational status/unemployment	23

Study design	Study period/ Countries	Sample size/ Age	Case number	Outcome assessment	Pooled risk estimates (95%CI)	Confounders	Reference
						rate, health insurance, per capita/household income, number of people in the household, environmental tobacco smoke, traffic noise annoyance ozone, heating with indoor combustion, workplace exposure with dust/fumes or organic solvents, type of heating energy, type of cooking energy, extreme temperatures, relative humidity, neighborhood socioeconomic index, census track data on median household income, percent of households living below the poverty line, neighborhood level recent immigrants, population density, prevalence of age≥65, latitude, urban residency, state of residence, region, urbanity, season, calendar year	
2 cohort, 5 time-series, 5 case-crossover	1984-2005 Canada, US, Italy, Denmark, China	11,120,555 ≥25 years	587,415	Death certificates, mortality data from hospital admissions, administrative/mortality database	T2D mortality<br>*$PM_{2.5}$*<br>RR: 1.123 (1.036-1.217)<br>*PM_{10}*<br>RR: 1.008 (1.004-1.013)<br>*NO_2*<br>RR: 1.024 (1.007-1.041)<br>per 10 μg/m^3 increment<br>*O_3*<br>RR: 1.065 (1.017-1.115)<br>per IQR increment	Age, sex, BMI, waist-to-hip ratio, physical activity, hypertension, hypercholesterolemia, fruit/vegetable consumption, fat consumption, smoking status and pack-years, alcohol consumption, educational level, unemployment rate, per capita income, workplace exposure with dust/fumes, environmental tobacco smoke, seasonal, sub-seasonal variations, meteorological conditions, days of the week, temperature, humidity, pressure, calendar year	[34]
6 cohort, 6 case-crossover, 5 time-series	1984-2006 Canada, US, Italy, Denmark, Germany, China	4,924,514 ≥21 years	1,254,982	Hospital discharge for T2D, self-reported diabetes, doctor-diagnosed T2D, elevated FBG or RBG levels, elevated plasma glucose levels after OGTT, administrative/mortality database	T2D morbidity/mortality<br>*$PM_{2.5}$*<br>RR/MRR: 1.05 (0.99-1.10)<br>*PM_{10}*<br>RR/MRR: 1.01 (1.003-1.013)<br>*NO_2*<br>RR/MRR: 1.04 (1.01-1.08)<br>*O_3*<br>RR/MRR: 1.07 (1.05-1.09)<br>*SO_2*<br>RR/MRR: 1.05 (1.012-1.079)<br>*SO4*<br>RR/MRR: 1.04 (1.02-1.079)	Age, sex, race, BMI, waist-to-hip ratio, hypertension, family history of diabetes, hypercholesterolemia, previous myocardial infarction, physical activity, fruit consumption, fat consumption, diet, smoking intensity, alcohol consumption, marital status, educational level, occupational level, environmental tobacco smoke, heating with fossil fuels, workplace exposure with dust/fumes, extreme temperatures, neighborhood socioeconomic index, season, calendar year, time, population changes/density, meteorological conditions, days of the week temperature, humidity, barometric pressure	35

Abbreviations: BMI: body mass index; 95%CI: 95% confidence interval; COPD: chronic obstructive pulmonary disease; FBG: fasting blood glucose; HbA1c: hemoglobin A1c; HR: hazard ratio; hsCRP: hypersensitive C-reactive protein defined as CRP level > sample median(1.0mmol/l); IQR: interquartile range; MRR: mortality rate ratio; NO_2: nitrogen dioxide; NO_x: nitrogen oxides; O_3:: ozone; OGTT: oral glucose tolerance test; OR: odd ratio; $PM_{2.5}$: fine inhalable particles, with diameters that generally 2.5 micrometers and smaller; PM_{10}: inhalable particles, with diameters that are 10 micrometers and smaller; RBG: random blood glucose; RR: relative risk; SO_2: sulfur dioxide; SO_4: sulfate T2D: type 2 diabetes
